# Facilitators and barriers to tuberculosis active case findings in low- and middle-income countries: a systematic review of qualitative research

**DOI:** 10.1186/s12879-023-08502-7

**Published:** 2023-08-07

**Authors:** Melkie Dagnaw Fenta, Oluwaseun Adeolu Ogundijo, Ahmed Abi Abdi Warsame, Abebaw Getachew Belay

**Affiliations:** 1 Department of Clinical Veterinary Medicine, University of Gondar, Gondar, Ethiopia; 2 Department of Veterinary Public Health and Preventive Medicine, University of Ibadan, Ibadan, Nigeria; 3 Department of Animal Production and Marketing, Faculty of Agriculture and Environment Science, Gulu University, Gulu, Uganda; 4 Department of Veterinary Public Health and Epidemiology, University of Gondar, Gondar, Ethiopia

**Keywords:** LMICs, Tuberculosis, Facilitators and barriers, Active case finding, Systematic review

## Abstract

**Background:**

Tuberculosis (TB) is an ancient infection and a major public health problem in many low- and middle-income countries (LMICs). Active case finding (ACF) programs have been established to effectively reduce TB in endemic global communities. However, there is little information about the evidence-based benefits of active case finding at both the individual and community levels. Accurately identifying the facilitators and barriers to TB-ACF provides information that can be used in planning and design as the world aims to end the global TB epidemic by 2035. Therefore, this study aimed to identify the facilitators and barriers to tuberculosis ACF in LMICs.

**Methods:**

A systematic search was performed using recognized databases such as PubMed, Google Scholar, SCOPUS, HINARI, and other reference databases. Relevant studies that assessed or reported the ACF of TB conducted in LMICs were included in this study. The Joanna Briggs Institute’s (JBI) Critical Appraisal Tool was used to assess the quality of the selected studies. The Statement of Enhancing Transparency in Reporting the Synthesis of Qualitative Research (ENTREQ) was used to strengthen the protocol for this systematic review. The Confidence of Evidence Review Quality (CERQual) approach was also used to assess the reliability of the review findings.

**Results:**

From 228 search results, a total of 23 studies were included in the final review. Tuberculosis ACF results were generated under two main themes: barriers and facilitators in LMICs, and two sub-themes of the barriers (healthcare-related and non-healthcare-related barriers). Finally, barriers to active TB case finding were found to be related to (1) the healthcare workers’ experience, knowledge, and skills in detecting TB-ACF, (2) distance and time; (3) availability and workload of ACF healthcare workers; (4) barriers related to a lack of resources such as diagnostic equipment, reagents, and consumables at TB-ACF; (5) the stigma associated with TB-ACF detection; (6) the lack of training of existing and new healthcare professionals to detect TB-ACF; (7) communication strategies and language limitations associated with TB ACF; and (8) poor or no community awareness of tuberculosis. Stigma was the most patient-related obstacle to detecting active TB cases in LMICs.

**Conclusion:**

This review found that surveillance, monitoring, health worker training, integration into health systems, and long-term funding of health facilities were key to the sustainability of ACF in LMICs. Understanding the elimination of the identified barriers is critical to ensuring a maximum tuberculosis control strategy through ACF.

**Supplementary Information:**

The online version contains supplementary material available at 10.1186/s12879-023-08502-7.

## Introduction

### Background

Tuberculosis (TB) is a widespread and chronic infectious disease that is globally spread and is caused by the pathogen Mycobacterium tuberculosis [1]. In accordance with estimations, approximately 40 million individuals are afflicted with TB in the year 2022, with 3.5 million of those being children [2]. Low-and middle-income countries (LMICs) bear a disproportionate burden of high morbidity and mortality associated with TB [3]. The Southeast Asia and Western Pacific regions accounted for the highest number of TB cases, followed by the African region. In these regions, the morbidity rates can reach as high as 95%, with mortality rates up to 98% [4]. Tuberculosis case-finding mechanism is mostly passive in low- and middle-income countries due to the presence of potential obstacles. This system relies on individuals reporting for diagnosis rather than active outreach programs [5, 6].

Active case detection is a novel methodology for tuberculosis screening that exhibits significant potential to augment the timely identification of cases in underserved communities [7]. This approach is largely directed towards demographic groups that are considered high-risk, comprising individuals who are homeless, incarcerated, receiving care in nursing homes, and residing in economically disadvantaged regions [8]. Unlike passive case-finding, ACF involves actively searching for TB in individuals who would not seek care spontaneously. The strategy aimed to eradicate tuberculosis, as acknowledged by the World Health Organization, recognizes ACF as a vital methodology for the identification of tuberculosis cases that are currently being disregarded by healthcare facilities [9].

In nations like the United States, Northern America, Canada, and the European Union, where the occurrence of tuberculosis is low, there are policies in place that aim to proactively identify incidences of the disease. Conversely, a majority of lower and middle-income countries with substantial TB burdens depend on passive case detection. This reliance on passive case detection has contributed to the current failure to prevent transmission at the necessary level [10, 11]. Despite the potential that ACF holds for amplifying the early detection of cases among marginalized populations, there exist substantial barriers to the adoption of ACF strategies in low and middle-income countries. These barriers may include inadequate capacity-building for healthcare workers, limited accessibility to healthcare facilities, and insufficient community involvement [12]. Moreover, while some studies have investigated ACF interventions, there is a lack of summarized results about the barriers and facilitators of ACF.

This systematic review is necessary to provide evidence-based recommendations for ACF interventions that can increase the detection of TB cases in low and middle-income countries. The findings of this study will have significant implications for policymakers, healthcare professionals, and researchers, enabling them to plan and design effective TB ACF interventions that improve TB control in these countries. In addition, this investigation will contribute to the global efforts aimed at achieving the WHO End TB strategy and the sustainable development goals.

The present review highlights the significance of addressing the challenge of active case finding for tuberculosis (TB-ACF) in low- and middle-income countries (LMICs) through thorough investigation and evidence-based proposals as evidenced by the existing literature.

While it is acknowledged that TB remains a major public health challenge worldwide, with LMICs bearing a disproportionate burden of high morbidity and mortality, the current passive case-finding mechanism in these countries is insufficient to effectively identify and treat cases [10, 12].

The purpose of this study is to investigate the barriers and facilitators of TB-ACF in LMICs, providing insights and recommendations for future ACF policy development. By conducting a systematic review of existing studies, this research aims to fill the gap in summarized results and generate evidence-based findings that can guide policymakers, healthcare professionals, and researchers in planning and designing effective ACF interventions. The implications of this study are significant, as it has the potential to improve TB control in LMICs by enhancing case detection rates. The findings will inform the development of targeted interventions and strategies that overcome the identified barriers and leverage the facilitators of TB-ACF. Policymakers can use these recommendations to implement evidence-based policies that support proactive case identification, ultimately reducing transmission rates and improving patient outcomes.

Furthermore, this investigation aligns with global efforts to achieve the World Health Organization’s End TB strategy and the sustainable development goals. By focusing on TB-ACF in LMICs, where the burden of the disease is particularly high, this research contributes to the broader objective of eliminating TB as a global public health threat.

In summary, this systematic review is essential to justify the need for investigating TB-ACF in LMICs. By identifying barriers, facilitators, and providing evidence-based recommendations, this study aims to improve ACF policy development, enhance case detection rates, and contribute to global efforts in combating TB.

### Objective

To identify the facilitators and barriers of tuberculosis to ACF in low- and middle-income countries (LMICS).

### Review questions

- What are the facilitating factors for TB -ACF in low and middle-income countries?
- What are the barriers to active TB case detection in low- and middle-income countries?
- What are the healthcare system and non-healthcare system -related barriers to TB-ACF in LMICS?

### Methodology

#### Synthesis methods

Enhancing transparency in reporting the synthesis of qualitative research (ENTREQ) statement was used for strengthening the protocol for the systematic review [13] (Additional file 2).The items have been meticulously collated and systematically classified into five distinct categories, namely: introduction, methods and methodology, literature search and selection, appraisal, and synthesis of findings.

#### Inclusion criteria

The current systematic review included studies with any qualitative study design, which can be conducted using either qualitative studies or mixed methods. Criterion (1): research question the paper is based on a clearly defined research question, which is clearly discussed and referenced throughout the paper. Criteria 2: Internal validity: the design of the study is suitable for the posed research inquiry and has unambiguously enunciated the study objectives. Selection bias has been minimized; confounding factors have been identified and/or controlled; explanatory variables are based on sound scientific principles; and outcome measures are complete and reliable. Criteria 3: Clarity of Results: Well-described and appropriate analytical methods were used. The precision of association is given or calculable and is meaningful. Criteria 4: External validity: The source population is well described, and the eligible population represents the source population. Selected participants represent an eligible population, and the results are consistent with results from other studies. The study results are generalizable to the source population. Any study that used qualitative methods of data collection (individual interviews, focus group discussions, and observation) and data analysis (thematic analysis) was included. The articles examining barriers and/or facilitators have been included. Healthcare providers involved in active case finding, community health workers (HCWs) and volunteer health managers, peer volunteers, policymakers, suspected TB patients, activists, academics, and other stakeholders encountered in the studies on ACF were included in the review. All studies conducted in WHO Member States grouped into low- and middle-income countries were included. Articles on barriers and promoters examining factors related to active TB case finding at the health system, individual, and community levels in low- and middle-income countries were included.

#### Exclusion criteria

Study types like clinical trials, case-control, and cohort study types were excluded, and studies with comments from quantitative surveys, editorials, and opinion pieces were also excluded. Studies conducted within groups in high-income countries were also excluded. Studies not reported in English were excluded.

### Search strategy and data sources

**Fig. 1:**
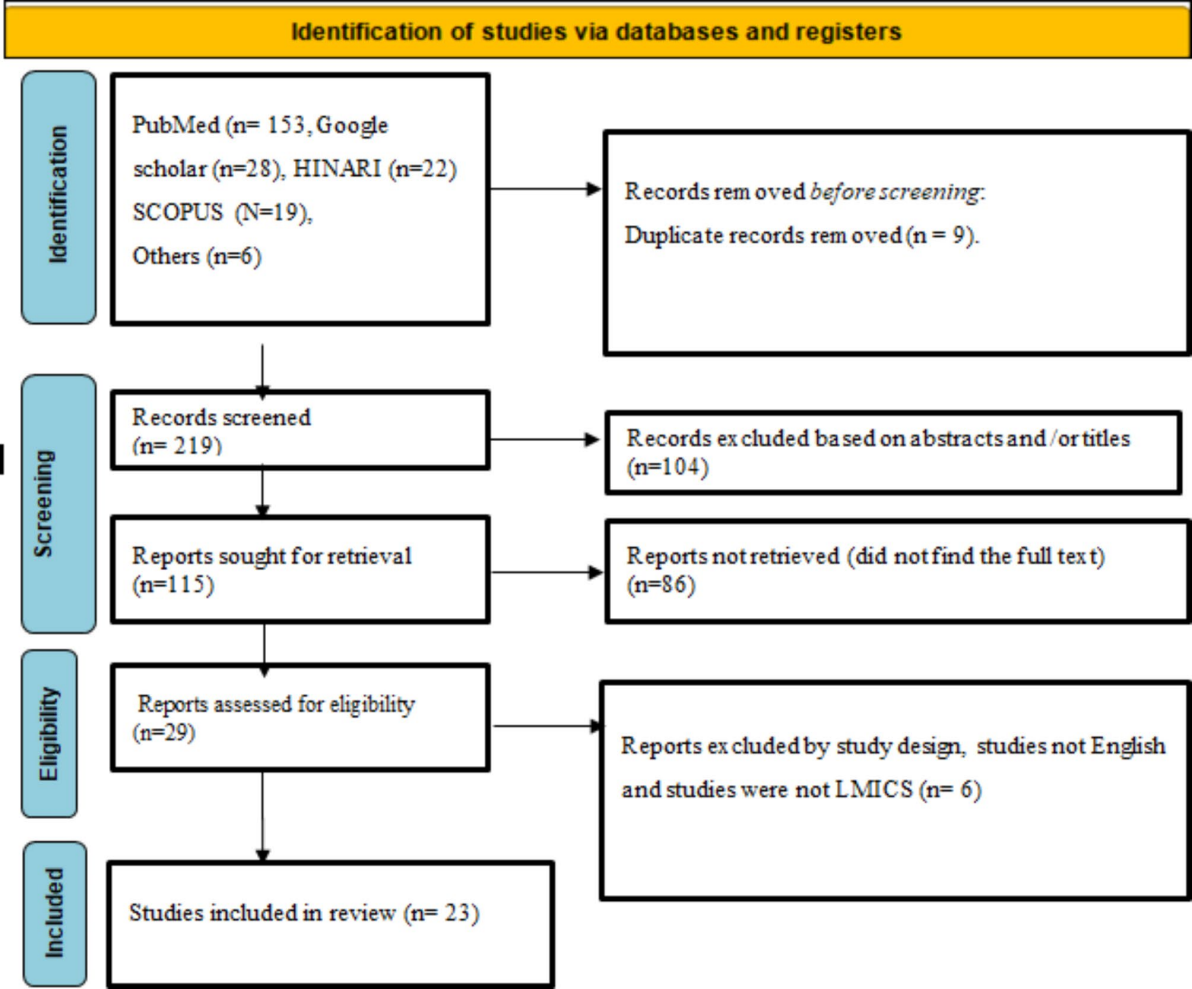
PRISMA flow diagram to investigate the facilitators and barriers of TB ACF in LMICs.

Electronic databases, including PubMed, Google Scholar, Scopus, HINARI, and other sources, were searched using Endnote Manager for sorting and filtering the articles for review. In addition, Microsoft Excel was used to facilitate the screening of the imported articles. The search strategy incorporated the key terms of the review question and utilized Boolean search operators. The key terms were: facilitators, enablers, barriers, challenges, “active case finding” “systematic screening” “community-based case finding” OR “community-based case detection” “tuberculosis,“ and “low and middle-income countries”. The search results were presented in the form of a flow diagram, as recommended by the Preferred Reporting Items for Systematic Reviews and Meta-analysis (PRISMA) (Fig. 1). A total of 228 articles were browsed through these electronic databases and other methods. A total of 9 articles were removed for duplicates, being marked as ineligible, or for other reasons. A total of 104 articles were excluded through title and abstract screening. One hundred fifteen (115) article reports were sought for retrieval, and 29 were evaluated for eligibility. Finally, only 23 full-text articles were deemed suitable for qualitative and quantitative synthesis.

### Quality assessment

The included relevant studies were critically assessed by two independent authors (AA and MD). The JBI Critical Appraisal Tool was used to assess the quality of the qualitative studies included in this review [14] (see additional file 1). In addition, we graded the final synthesized qualitative findings according to the CERQual approach to rate the level of confidence and certainty of the findings [15].

### Data extraction and analysis

In brief, the entirety of the retrieved publications in various databases underwent initial screening. The abstracts of the aforementioned publications were scrutinized by two independent reviewers (MD and OA) acting individually. Instances, where judgments conflicted, were subsequently resolved by the authors who made the initial screening decisions. The screening of full texts was executed in a comparably to that of abstracts. Then they were shared online with another reviewer (AA). The reviewers used a customized JBI data abstraction format. As members are located in different countries, online Zoom meetings were scheduled once a week for this process. For urgent purposes, phone calls, emails, and internet account group SMS were used as means of contact. These communications should discuss solutions to unclear points regarding the eligibility criteria and other confusing issues. For example, there was a disagreement between two reviewers (AA and MD) regarding the classification of barriers and promoters in low- and middle-income countries. This was resolved during one of our online engagements that all group members attended. We developed a standardized form to identify included studies and settings (publication year, published journals, study design, interventions, and country) and participant characteristics (type of participants, e.g., health workers, number of participants, etc.) and the primary outcome measures (key barriers and facilitators) in LMICs.

After conducting data extraction for each paper, the studies were categorically grouped according to the outcomes of interest. Subsequently, narrative summaries for each outcome were presented and analyzed. The data was then analyzed and summarized using both pictorial and tabular representations. Firstly, the findings were classified into two categories, namely, barriers related to low- and middle-income countries. Secondly, the theme was summarized into the main findings. Subsequently, the quality assessment tool was applied. Concurrently, the findings, especially the barriers, were categorized into theme 1 as healthcare system-related and non-healthcare system-related barriers. Finally, patient-related, health-related, resource-related, and implementation-related factors were considered. Ultimately, the quality assessment tool CERQual was applied to the findings. Six out of the total eight preliminary findings have been chosen as the “key findings” to undergo further analysis through the Confidence in the Evidence from Reviews of Qualitative Research (CERQual) tool. The selection process was based on a consensus between three authors (AG, AA), where the strength of evidence, number of supporting reviews, level of variability in review findings, and significance of the findings as indicated in the included reviews were taken into consideration.

## Results

### Review description

A total of 23 studies from 2013 to 2020 were included in the final review. These studies were organized according to the title of the article, study location or country, methods, sample characteristics, and author(s) with the year of publication (see Table 1). The results were presented based on the major themes of barriers and facilitators that emerged from the analysis and synthesis. The barriers are further presented as healthcare system and non-healthcare system related barriers. Of the included studies, it was noted that two of the articles in question did not explicitly mention the ethical considerations that were taken into account when formulating their research protocols.

**Table 1:** Summary of study characteristics (n = 23)

Title of the article	Study Location	Methods	Ethical Approval	Participants	Settings	Data collection	Author
Identifying barriers to and facilitators of TB contact investigation in Kampala	Uganda	Qualitative	Not specified	HCW from seven health centers	Clinic-based health staff, clinic-affiliated LHWs, and adult household contacts of index patients	Group discussions and interviews	[16]
Experts’ insights into the development and implementation of active tuberculosis case-finding policies globally	Nepal	Exploratory qualitative	Yes	Female Community Health Volunteers and TB-infected people	Four districts of Nepal with a high prevalence of poverty and TB	Semi-structured and key-informant interviews	[17]
Analysis of factors that influence early TB case detection among aged 15 years and above	Liberia	Qualitative	Not specified	Senior directors, managers of National Leprosy and TB Control centers	15 years and above in Liberia	Internet	[18]
Patient and community experiences of tuberculosis diagnosis and care within a community-based intervention in Ethiopia: a qualitative study	Ethiopia	Qualitative	Yes	Clients of the community-based intervention	Treatment-seeking behavior and Perceptions of the Community Six districts	In-depth interviews	[19]
Barriers for tuberculosis active case finding	Ethiopia	Qualitative	Yes	TB treatment providers, program managers and TB patients	Governmental health facilities, urban health centers and rural health centers	In-depth interviews	[20]
Peer-led active tuberculosis case-finding among people living with HIV	Nepal	Peer-led method and clinical observation	Yes	Peer volunteers	Community districts	Screening tools and Clinical diagnosis	[21]
A yield and cost comparison of TB contact investigation and intensified case finding	Uganda	Qualitative	Yes				[22]
Challenges from tuberculosis diagnosis to care in community-based active case finding among the urban	Cambodia	Mixed-Methods	Yes	TB-infected village volunteers and health workers	Poor urban settlements	In-depth interviews and cross-sectional survey	[23]
Enablers and challenges in the implementation of active case findings in a selected district.	India	Qualitative	Yes	Healthcare providers	TB diagnostic units of Bengaluru rural district	In-depth interviews	[24]
Barriers to the access, diagnosis, and treatment completion for tuberculosis patients	Nepal	Qualitative	Yes	Patients, traditional healers, community members, and healthcare workers	Tanahuh, Kaski, Parsa, Nawal parasi, Mustang and Kathmandudistricts in Nepal	In-depth interviews, focus group discussions, and semi-structured interviews	[25]
Exploration of barriers and facilitators to household contact tracing of index tuberculosis cases	Ethiopia	Descriptive qualitative	Yes	HEWs, index TB patients, household contacts of TB patients, health center TB focal and district TB coordinators	Alamo District	In-depth and key informant interview	[26]
Turning off the tap: stopping tuberculosis transmission through active case-finding and prompt effective treatment.	Pakistan	RE-AIM strategy	Yes	Mobile unit attendees	Three-district region of Lima, Peru	In-depth interviews, Chest radiography	[11]
Improving active case finding for tuberculosis	South Africa	Qualitative	Yes	TB patients and community members	Vhembe and Waterberg districts	Semi-structured, in-depth interviews and focus group discussions	[27]
Patient-led active tuberculosis case-finding	Democratic Republic of Congo(DRC)	Patient-led strategy	Yes	Volunteer patients	DRC selected districts	Family history clerking	[28]
Optimizing tuberculosis contact investigation and linkage to care in Nairobi	Kenya	Multi-method qualitative	Yes	TB patients and healthcare workers	Study sites in Nairobi	Individual interview, focus group discussions, and key informant review	[29]
Capitalizing on facilitators and addressing barriers when implementing active tuberculosis case-finding in six districts of Ho Chi Minh City	Vietnam	Exploratory qualitative	Yes	Community members, healthcare staff and volunteers	Ho Chi Minh City districts	Semi-structured and key-informant interviews	[30].
Active TB case finding in a high burden setting; comparison of community and facility-based strategies	Zambia	Mixed-Method	Yes	Community healthcare workers	Peri-urban settlements in Lusaka district	Digital chest x-ray, sputum analysis, and community engagement via symposia and media communications	[31]
Developing strategies to address barriers for tuberculosis case finding and retention in care among refugees in slums	Uganda	COMB-B model	Yes	Health care workers, community leaders, refugee TB patients and caregivers of TB patients	Urban slum in Kampala City	Key informant and in-depths interviews	[32]
Factors associated with DELAY in diagnosis among tuberculosis patients in Hohoe Municipality	Ghana	Mixed	Yes	New TB patients	Healthcare facilities at Hohoe Municipality	Patients’ records and interviews	[33]
Barriers to tuberculosis case finding in primary and secondary health facilities in Ghana: perceptions, experiences and practices of healthcare workers	Ghana	Qualitative	Yes	Healthcare workers	Rural health centers	Clinical observations and in-depth interviews	[34]
Factors affecting tuberculosis health message recall 2 years after active case finding in Blantyre	Malawi	Mixed-methods	Yes	Community peer group and TB monitoring officers	Urban slums of Blantyre	In-depth interviews and focus group discussions	[35]
Facilitators and Farriers to the implementation of a childhood tuberculosis control program	Bangladesh	Triangulation Design and Mixed	Yes	Policymakers, program managers, healthcare workers, and consumers	Urban and DOTS centers	In-depth interviews and key informant interview	[36]
Factors influencing the implementation of TB screening among PLHIV in selected HIV clinics	Ghana	Qualitative	Yes	HIV care providers	Regions, districts and facilities with TB/HIV coordinators	Indepth interviews and focus group discussions	[37]

### Healthcare system and governmental related barriers

The challenges in implementing ACF in healthcare have been inadequate healthcare worker training and staff shortages. The main gaps in tuberculosis control have been recognized as the limited availability of healthcare facilities and insufficient community involvement [12, 38]. Funding subsistence for tuberculosis care is a long-term challenge in many countries. To alleviate this financial burden, specific measures are needed at different levels, including linking tuberculosis to the overall social protection system [26, 39]. Resource constraints in LMICs limit TB contact investigation despite its benefits outweighing its cost and its increased efficiency when compared with intensified case finding (see Tables 2 and 3). Inadequate laboratory infrastructure for maintaining functional Xpert equipment further challenges implementation and scale-up [40]. Generally, shortages of healthcare providers, inadequate basic infrastructure, and inadequate diagnostic equipment and supplies were identified as barriers to TB case finding [41]. Also included was limited access to TB diagnostic services, which can be absent or characterized by delays in the diagnostic process.

Access and health service delays, longer distances, transportation costs, poor quality of services, understaffing, poor motivation, outdated protocols, limited laboratory supplies, limited screening among high-risk groups, and poor data quality and feedback systems were the major constraints to TB diagnosis and implementation of services in Liberia [42]. Commonly noted barriers in Uganda included insufficient time and space in clinics for counseling and mistrust of health-center staff among index patients and contacts [43]. Logistics and infrastructure, waiting time and institutional readiness, referral, feedback, human resources, charges for using some laboratories, workloads, and distance to TB facilities were barriers to TB contact tracing and investigation in Ethiopia [44].

**Table 2:** Key barriers and facilitators of active TB case findings in low-income countries identified from the included studies (n = 11)

Study location	Facilitators	Barriers	Authors
Ethiopia	Social support and training of health workers	Distance, shortage of money, stigma, discrimination, the workload of household contacts and health workers, shortage of reagents, absence of well-ventilated TB class, and the lack of regular monitoring and supervision	[26]
Uganda	Education, persuasion, enablement, modeling of health-positive behaviors, incentivization,and restructuring of the health service environment	Stigma, limited knowledge about TB among contacts, insufficient time and space in clinics for counseling, mistrust of health center staff among index patients and contacts, and high travel costs for LHWs and contacts	[16]
Liberia	Develop a TB communication strategy, strengthen community-based DOTs, and intensify screening, knowledge and awareness of TB	No regular monitoring of household contacts, screening for index TB cases, longer distances, transportation cost, stigma, misconception about TB signs, outdated protocols, limited laboratory supplies, poor data quality and feedback system	[18]
Ethiopia	Embedding TB services within communities was an acceptable approach for vulnerable groups experiencing poor access to health facilities.	Difficulties faced in accessing district-level health facilities	[19]
Ethiopia		Inadequate resources, limited access to diagnostic services, and inadequate diagnostic equipment and supplies	[20]
Uganda	The ability of LHWs to persuade index patients, communicate with patients via mobile phones, trust between index patients and LHWs, flexible scheduling of home visits, evaluation, the reduce risk associated with the transport of LHWs, family social support for contacts and payment for LHWs.	Lack of local contact investigation guidelines, lack of travel funds for contacts, stigma, lack of TB knowledge among contacts, the language barrier between LHWs and contacts, difficulties in locating households, avoidant behaviors of contacts, and fear of TB diagnosis among contacts	[22]
Kenya	Invitation of TB patients to bring contacts and HWS close and patients understanding the transmission of TB as a proactive measure by HWS	Long waiting times, inconducive clinic hours, poor community awareness about TB, and stigma.	[29]
DRC	Peer-led increase in active TB cases finding		[28]
Zambia	Patients screened at the facility level, increased awareness and demand creation activities, and the use of more sensitive screening and diagnostic tools	Patients screened from the general community and long waiting time	[31]
Uganda	Physical capability (availability of free TB services in public health facilities), social opportunity (availability of translators), identified education, incentivization, and training	Unavailability and easily accessible private facilities with no capacity to diagnose and treat TB in the community, lack of knowledge about TB among refugees, and widespread of TB stigmatization and language barrier, poor living conditions, mobility of refugees, lack of facilitation for health workers, discrimination and rejection of TB patients	[32]
Malawi	Community education, sensitization and engagement, community need to ACF when service is at their home	Fear of HIV diagnosis and association, health care seeking behavior barriers	[35]

**Table 3:** Key barriers and facilitators of active TB case findings in middle income countries identified from the included studies (n = 12)

Study location /Country	Facilitators	Barriers	Authors
Cambodia	Build trust and facilitate communication, a patient-centered approach and community involvement	High indirect costs, privacy and stigma issues, and anticipated treatment side effects	[23]
Nepal	The utility of education for providers and appointments in which physicians use Xpert in TB diagnosis was noted to have improved the acceptability	Re-evaluation by pulmonologists at the government hospitals, even if they had positive Xpert, and the lack of knowledge about diagnostic tests	[17]
South Africa	Door-to-door activities giving access to respected leaders of the community, such as chiefs and civic leaders; Incentivization	Lack of TB knowledge, social (TB stigma), and structural factors (distance, time and lack of money for transportation)	[27]
India	Involving local leaders and panchayat members, issuing identity cards to field staff, increasing monetary incentives, training ASHA in counseling and sputum collection, and financial support to patients for chest X-ray examination and travel	Inadequate training, shortage of staff, stigma, lack of awareness about TB, illiteracy, inability to convince patients for sputum tests, and delay in getting CBNAAT.	[24]
Nepal		Lack of trained health personnel, lack of equipment ,and irregular presence of health workers	[25]
Pakistan	The inclusion of targeted active case-finding in a comprehensive epidemic-control strategy for tuberculosis		[11]
Vietnam	Communication and awareness-raising, preparation and logistics, data systems and processes, and incentives. Strengths of employees and volunteers to capitalize on experience, skills, and communication.	Stigma, discrimination, and mistrust	[32]
Ghana		Lack of medical insurance, perceived stigma, and making multiple healthcare encounters.	[33]
Ghana		Health system barriers include lack of TB diagnostic laboratories in rural health facilities and no standard referral system to the municipal hospital for further assessment and TB testing. Heath worker- related barriers such as lack of training on case detection guidelines, fear of infection (exacerbated by lack of appropriate personal protective equipment and lack of motivation among HWS for TB work	[34]
Bangladesh	Government stewardship, presence of specific guidelines, knowledge and capacity building of frontline health workers	Lack of diagnostic facilities, lack of diagnostic facilities, and poor engagement of private practitioners	[36]
Ghana	Good communication and referral channels, health workers recognizing the need for interventions and the role of chemical sellers	Low commitment of the implementers to screen for TB.	[37]

### Healthcare system-related and non- healthcare system-related barriers

**Fig. 2:**
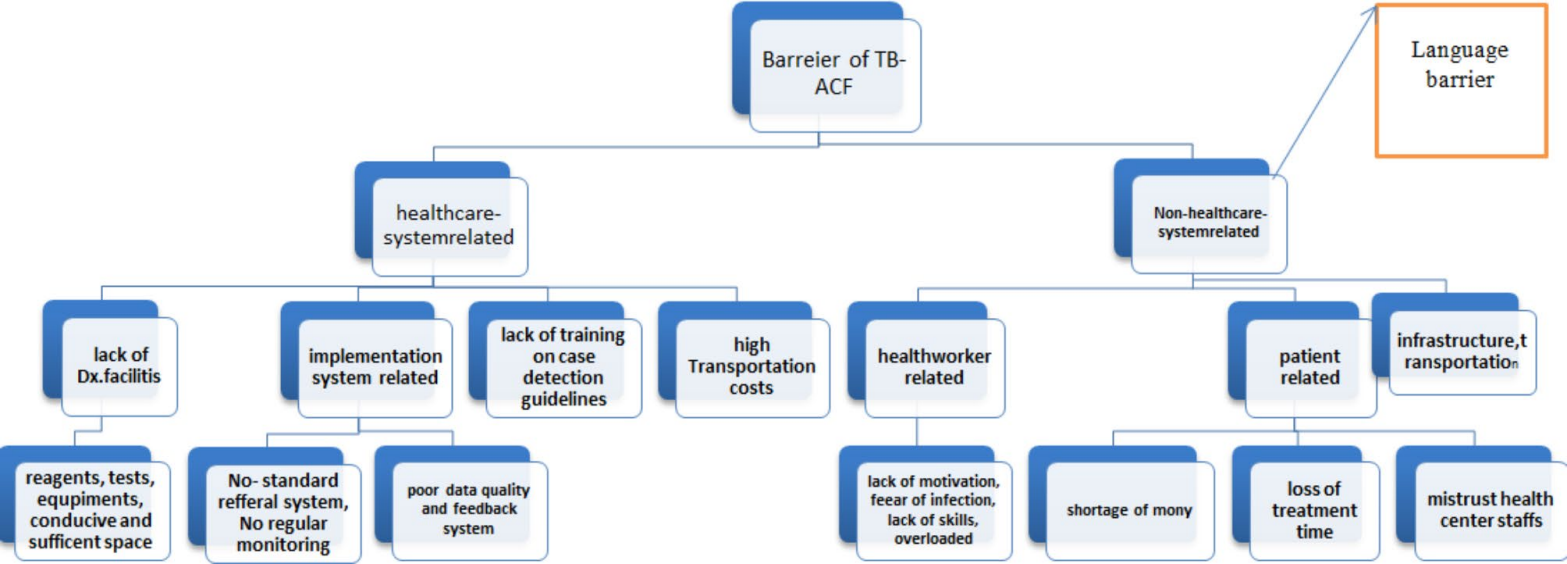
The major barberries classified under healthcare and non-healthcare systems in LMICS

The identification of active tuberculosis is impeded by two main categories of obstacles, namely health system-related and non-health system-related barriers, as illustrated in Table 4; Fig. 2. The studies analyzed in this research revealed several health system-related barriers, among which a dearth of diagnostic resources (including equipment, reagents, and tests), as well as inadequate TB awareness, were the most frequently observed ones. Conversely, barriers not associated with the healthcare system encompassed stigmatization, transportation costs, insufficient education, limited awareness concerning diagnostic testing, and the presence of a language barrier.

Limited understanding and awareness regarding the identification of signs and symptoms, as well as misconceptions surrounding tuberculosis, have proven to be the primary contributing factors to the delay in patients accessing tuberculosis services [44]. Nevertheless, the familiarity and knowledge of the community demonstrated by village health volunteers have been crucial in facilitating the initial access to active case-finding participants. However, at times, this familiarity has negatively impacted their perceived legitimacy among community members who are aware of their lack of medical training. To gain respect among their peers in the community, village health volunteers recognize the importance of affiliating with tuberculosis workers and other trained healthcare providers [45].

According to Marangu et al.[29], the key barriers to CI were the failure of HCWs to educate and invite TB patients to bring close contacts for TB screening, sub-optimal processes and flow of TB patients, HCW and community TB-related stigma [46]. In another study, knowledge, commitment and motivation, and stigma and discrimination for household contact-tracing of index TB cases were also identified [47]. One practical consideration for the implementation of targeted ACF activities was convincing individuals to undergo screening for a stigmatized disease, especially if the screening requires time and effort on the part of the individuals[24]. Seeking private health care and self-medication before TB diagnosis, lack of perceived risk, threat, susceptibility, and stigma derived qualitatively further explained the quantitative findings [41]. The different attitude of the community due to stigma, lack of awareness about TB, illiteracy and inability to convince patients for sputum tests were challenges to conduct ACF [42]. High travel costs for LHWs and contacts were also noted as barriers to TB contact investigation [43].

**Table 4:** Healthcare system and non- healthcare system-related barriers to tuberculosis active case findings in LMICS (n = 21)

S. No	Barriers		Author
	Theme-1: Healthcare system related	Theme − 2: Non- healthcare system	
1		privacy and stigma issues ,anticipated treatment side effects, High indirect costs	[23]
2	Re-evaluation by pulmonologists at the government hospitals, even if they had positive Xpert, and	the lack of knowledge about diagnostic tests	[17]
3		Stigmatization	[21]
4		Stigma, time, Lack of TB Knowledge, Distance and lack of money for transportation	[27]
5	lack of awareness about TB, shortage of staff, Inadequate training	stigma, illiteracy, inability to convince patients to sputum test	[24]
6	lack of equipment	Lack of trained health personnel ,irregular presence of health workers	[25]
7	---------------------	Stigma, discrimination, and mistrust	[32]
8	Lack of medical insurance, making multiple healthcare encounters.	perceived stigma,	[33]
9	lack of TB diagnostic laboratories, no standard referral system to the municipal hospital for further assessment and TB testing, lack of training on case detection guidelines	fear of infection, lack of motivation among HWS for TB workers	[34]
10	lack of diagnostic facilities, and poor engagement of private practitioners	--------	[36]
11	The low commitment of the implementers to screen for TB	------	[37]
12	shortage of reagents, absence of well-ventilated TB class, the lack of regular monitoring and supervision and the workload on health workers,	Shortage of money, stigma, discrimination, workload of household contacts and distance	[16]
13	insufficient space in clinics, high travel costs for LHWs ,insufficient time	Stigma, limited knowledge about TB among contacts, mistrust of health center staff among index patients and contacts	[18]
14	limited laboratory supplies, No regular monitoring of household contacts, poor data quality and feedback system	stigma, screening for index TB cases &misconception about TB signs, transportation cost	[19]
15	Difficulties faced in accessing district level health facilities	---------------------------	[20]
16	limited access to diagnostic services, inadequate diagnostic equipment and supplies, as well asinadequate resources	---------	[16]
17		language barrier between LHWs and contacts, difficulties in locating households, avoidant behaviors of contacts, fear of TB diagnosis among contacts, lack of travel funds for contacts	[29]
18	Long waiting times, in conducive clinics, poor community awareness about TB	Stigma	[28]
19	Patients screened from the general community and long waiting time	----------------	[31]
20	Unavailability and easily accessible private facilities with no capacity to diagnose and treat TB in the community	language barrier, mobility of refugees, wide spread of TB stigmatization, lack of knowledge about TB among refugees	[32]
21	health care seeking behavior barriers	Fear of HIV Diagnosis and Association	[35]

### Facilitators

The most important facilitators identified were the personalized and enabling services provided by LHWs. The study identified education, persuasion, enablement, modeling of health-positive behaviors, incentivization, and restructuring of the service environment as relevant intervention functions with potentials to alleviate barriers to and enhance facilitators of TB contact investigations[26]. Implementers’ motivation and incentives fostered active case-finding policy implementation[48]. Public health providers were motivated by the increased early case detection and expedited sequence from screening to treatment. TB workers were motivated by the increased awareness, improved health-seeking behaviors among TB patients and strengthened competence of village health volunteers in target communities [49].

To address the challenge of TB “missing cases”, policies, effective strategies, and implementation of active case interventions for TB key populations are highly essential globally [17]. By bringing TB services closer to the community, participants reported that ACF had removed barriers to access and cost. This was particularly appreciated by the elderly and severely ill, whose physical conditions prevented them from traveling to the health centers for screening and treatment [50]. Ensuring and outlining good health outcomes, and limiting TB transmission within hard-to-reach groups, can prove challenging. The reason is that the hard-to-reach groups may experience difficulties in accessing care, and if they do access it, they may experience difficulties remaining in it[51].

Approaches such as improving TB diagnostic tools and algorithms, and engaging all care providers are suggested to find missing TB patients[52]. The involvement of HCWs in the general activities of counseling patients, and issuing of identity cards to them for ease of recognition, was recommended to foster ACF [53]. To maintain benefits from different approaches, there is a need to distinguish between CHWs that are trained and remunerated to be a part of an existing health system and those who, with little training, take on roles and are motivated by a range of contextual factors. Governments and planners can benefit from understanding the program that can best be supported in their communities, thereby maximizing motivation and effectiveness[54].

### Assessment of confidence in the evidence of the review findings

**Fig. 3:**
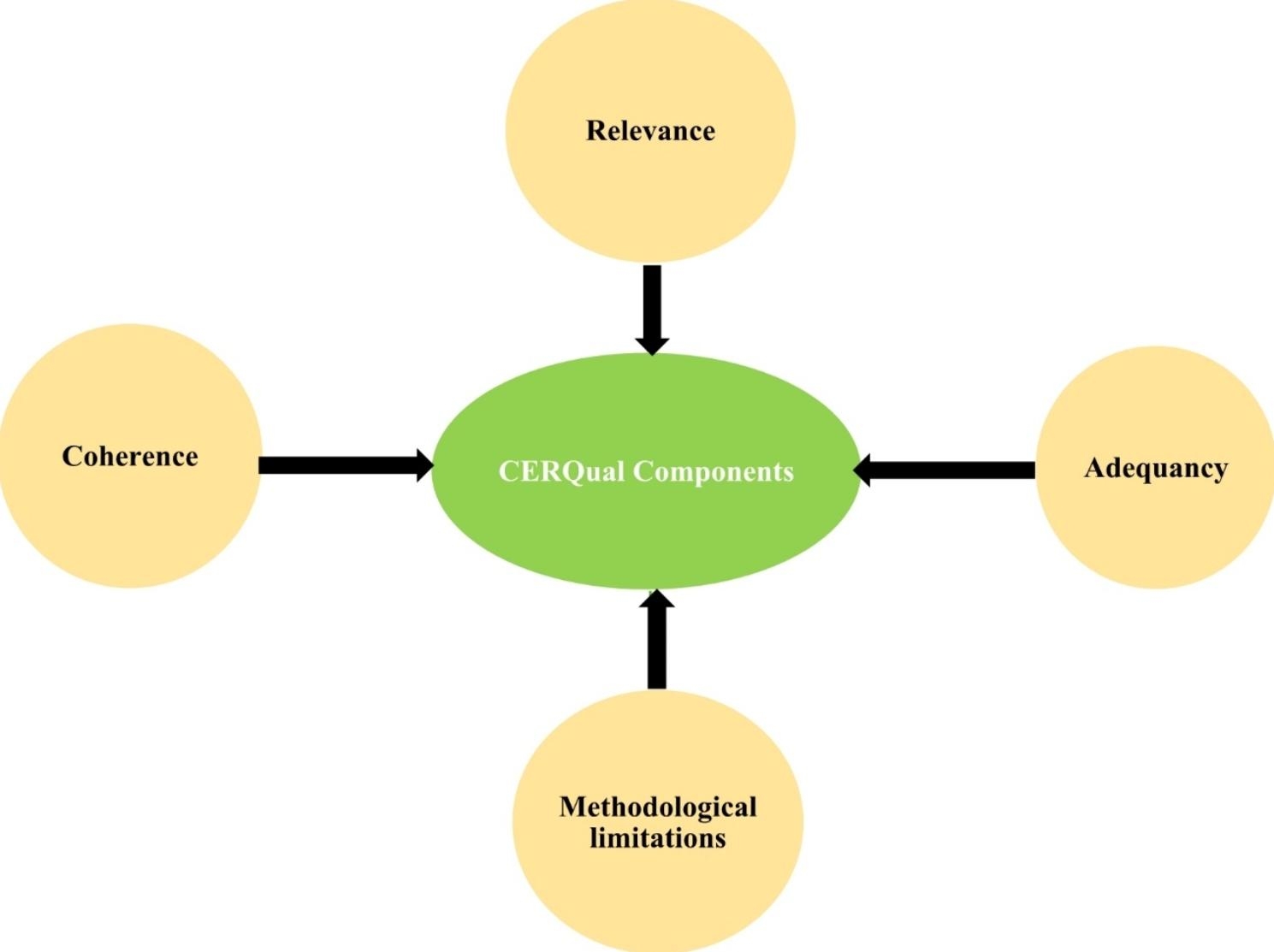
The components of CERQual

Two of the reviewer authors (MD and AG) applied the confidence of evidence review quality (CERQual) approach to assess the confidence in the findings of the review independently. Then, the evidence review quality approach assesses confidence in the review findings and is conducted based on four components namely; the methodological limitations of included studies, the relevance of the included studies to the review question, the coherence of the review findings and the adequacy of data contributing to the review findings [55] as shown Fig. 3. Every issue or phenomenon that was relevant to the four components was noted in the review results and taken into account when determining the overall CERQual assessment as high, moderate, low, or extremely low as depicted in Table 5.

The barriers that were identified during our review have been consolidated into eight overarching themes. These themes were derived from general concepts and through consensus among the review authors, as detailed in Table 6. In addition, our summary of qualitative findings included a CERQual assessment and a written justification, as outlined previously. It is important to note that the findings presented in our review are contingent upon various factors, such as the review question, the synthesis techniques employed, the intended application or target of the synthesis, and the depth of the data provided. These factors collectively influence the conclusions that we have drawn from our analysis [56].

**Table 5:** Summary of CERQual Confidence Rating on the main findings

Key findings	CERQual Rating	Explanation of the evidence assessment rating
Health workers encounter barriers pertaining about their experiences, knowledge, and skill set in identifying tuberculosis through active case finding, even when situated in healthcare establishments.	Moderate confidence	Although half of the articles raise important methodological issues (2/4), it is noteworthy that the principal discovery is consistent in all articles have significant methodological concerns, yet the key finding is consistently supported by directly relevant data in reviews with only minor
Direct and indirect costs of the patients	High confidence	All of the articles presented in this study provide supporting evidence for the primary findings. However, it is important to note that one (1/4) of articles have brought to light significant methodological flaws. In contrast, assessments that exhibit other methodological issues are can offer direct and valuable insights into both the primary findings
The present study investigates the accessibility and the magnitude of workloads that health workers face in the context of tuberculosis active case finding (ACF).	high confidence	The data evaluated in this investigation is of great significance (3/3) and has been thoroughly documented. The evaluations present unequivocal proof that is methodologically sound.
The lack of essential resources such as diagnostic equipment, reagents, and TB ACF supplies is a significant hindrance to effective tuberculosis control efforts.	Very low confidence	The methodology-related problems addressed by the present study relate to the indirect or partial applicability of earlier reviews that provided support for the primary finding.
Barriers related to stigma in the detection of TB ACF	Moderate confidence	In general, the included studies were moderately conducted (5/6). The review findings cut across many study settings and scientific procedures.
Barriers pertaining to the communication strategy and language limitations in the context of tuberculosis active case finding pose significant challenges.	Moderate confidence	The methodologies of the three studies were very critical and followed scientific procedures. Some of the studies have direct and indirect relevance.

**Table 6:** Confidence of Evidence Review quality (CERQual) summary of review findings, appraisal, and synthesis of qualitative research evidence on the barriers and facilitators to tuberculosis active case findings in low- and middle-income countries

S/N	key review findings	Contributing studies	Methodological limitation	Coherence		Adequacy		Relevance	Confidence in evidence	Explanation of the evidence assessment
1.	Barriers related to experiences, knowledge, and skills of health workers to detect TB ACF, even in health facilities	[17, 25–27]	Moderate concern	Very minor concern	Minor concern		Very minor concern		High	The data were from three middle and one low income countries. There are direct and indirect relevance in the studies also.
2.	Barriers related to distance and time, including funds related to transportation and incentives for the patients	[16, 23, 26, 27]	Very minor concern	Very minor concern	Very minor concern		Very minor concern		High	The methodologies of the three studies were very critical and follow scientific procedures. The studies had 50% distribution between low and middle income countries covering Ethiopia, Cambodia, South Africa and Uganda.
3.	Barriers related to the availability and workloads of health workers in TB ACF	[22, 24, 25]	Moderate concern	Very minor concern	Serious concern		Moderate concern		Very low	The methodological limitations of two of the studies were unclear study designs, inappropriate data collection technique and very thin data. The data was generated from low income country (Ethiopia) and middle income countries (Nepal and India).
4.	Barriers related to the absence of resources like diagnostic equipment, reagents and supplies for TB ACF	[17, 22, 25, 26, 36]	Moderate concern	Very minor concern	Serious concern		Serious concern		Very low	Some of the studies had direct and indirect relevance, very thin data, and lack ethical considerations. Data were collected from studies from low (Ethiopia and Uganda) and middle income countries (Nepal and Bangladesh).
5.	Barriers related to stigma in the detection of TB ACF	[16, 17, 21, 22, 26, 32]	Very minor concern	Very minor concern	Very minor concern		Very minor concern		Moderate	In general, the included studies were moderately conducted. The review findings cut across many study settings and scientific procedures. Data were sourced from four low (between Ethiopia and Uganda) and three middle (between Nepal and Bangladesh) income countries.
6.	Barriers related to lack of training for existing and new health workers on the detection of TBACF	[22,24, 25,29 ]	Very minor concern	Very minor concern	Very minor concern		Very minor concern			The methodology of the study was very critical and follows scientific procedures. The data sources were from low and middle income countries such as Ethiopia, Cambodia, South-Africa and Uganda
7.	Barriers related to communication strategy and language restrictions for tuberculosis ACF	[22, 32, 37]	Very minor concern	Very minor concern	Very minor concern		Very minor concern			The methodologies of the three studies were very critical and follow scientific procedures. Some of the studies have direct and indirect relevance. The data sources were from low and middle income countries such as Ethiopia, Cambodia, South-Africa and Uganda.
8.	Barriers related to inadequate or lack of community awareness about TB among contacts	[11, 16, 18]								In general, studies were moderately executed. However, the findings were sourced from 2 countries.

## Discussion

In this qualitative systematic review, we identified 23 studies (12 from middle-income countries and 11 from low-income countries), of which 19 reported qualitative data methods on barriers and facilitators of TB case finding to earlier detection in LMICs. All studies of these LMICs identified hard-to-reach communities as the main carriers of this disease. Advanced and effective TB programs are needed given the disease’s contagiousness, airborne transmission and rising TB incidence in some countries. In terms of the study design, five of the investigations delineated mixed-method barriers and facilitators [23, 31, 33, 36].

In general, the overall evidence supporting the factors influencing tuberculosis ACF in low- and middle-income countries were high at present, despite several interventions and varying types of tools adopted in different patient populations in the LMICs. This study reported a slightly positive influence associated with facilitators of TB contact investigation and ACF. There were a few overlapping explanations for the factors influencing TB ACF [16, 22–24, 26, 27, 32] and for positive psycho-social interventions used as the facilitators of ACF. In tuberculosis ACF, systematic identification and screening of people with presumptive TB symptoms, in a pre-determined target group, using tests, examinations, or other procedures that can be applied rapidly [57]. Interventions relating more to the psycho-social factors in persons, who were asymptomatic for TB, such as group-based psychotherapy, are important to boost and give confidence to stigmatized persons. This way could improve interpersonal and self-esteem as well as public health value by influencing positively psycho-social factors like mood, thereby affecting interpersonal barriers.

Inadequate resources, limited access to diagnostic services, inadequate diagnostic equipment and supplies were the most important factors influencing active case findings both low and middle-income countries [17, 20, 25, 36], while TB communication strategy and language were the key barriers for tuberculosis ACF [16, 18]. Peer-led, patient-centered approaches and community involvement increased the active finding of TB cases [23, 28].

This study suggested that TB case identification may be improved if the community could easily access health facilities with TB diagnostic services. Similarly, studies show that case notification increase in areas where community members have better access to facilities with TB diagnostic services [20, 58]. In most low income countries, health extension workers are intentionally placed at the community level to reinforce the accessibility of the rural population to different health services, including TB care. Still, health extension workers lack, so to alleviate the lack of transportation facilities to reach TB services and increase the ACF strategies the number of health extension workers and community-based identification methods should be scaled up in rural and remote areas.

The use of an active case-finding approach, which includes conducting household symptom screening through door-to-door community visits coupled with the integration of laboratory testing, has resulted in improved detection of tuberculosis cases in rural areas. This finding is supported by reference [59]. Moreover, active case finding through community outreach improved the speed of TB case finding, which indicated a possibility to reduce delays in TB diagnosis [60]. This strategy could help in solving problems related to unmet health needs in general, and undiagnosed TB cases in particular, for the rural population. The current review has ascertained that stigma is frequently encountered and functions as an impediment to obtaining care for those with ACF-TB; this may plausibly be attributed to the lack of health advocacy and awareness initiatives. Stigmatization and discrimination pose significant risks to individuals undergoing TB screening or diagnosed with TB, potentially undermining the success of the screening process.

The needs of vulnerable people must be served by increasing access to healthcare services while simultaneously minimizing the direct and indirect costs of seeking medical care. This can be achieved by strengthening primary health-care services, providing additional outreach services that cater to these populations, and implementing social protection schemes where necessary. In order to ensure that individuals with prevalent active TB cases seek care at facilities capable of diagnosing and treating TB, community engagement and demand in communities at a higher risk of TB must be increased. The implementation and adaptation of bidirectional screening, as demonstrated by this investigation, provides valuable proof to enhance the outcomes of TB-active case detection.

The findings of this qualitative systematic review have significant implications for policy modification and development in the context of tuberculosis active case finding (TB-ACF) in low- and middle-income countries (LMICs). The identified studies highlight various barriers and facilitators that influence TB case detection and earlier diagnosis in LMICs [21].

One key implication is the need for advanced and effective TB-ACF programs, considering the contagiousness and airborne transmission of the disease, as well as the rising TB incidence in certain countries. The study emphasizes the importance of targeted interventions and tools to reach hard-to-reach communities, which have been identified as the main carriers of TB [22].

The study also highlights the value of psychosocial interventions, such as group-based psychotherapy, to help stigmatized people overcome interpersonal barriers, increase their confidence, and boost their self-esteem. By influencing psychosocial aspects such as mood and promoting improved access to diagnostic services, these interventions can have a positive impact on TB-ACF outcomes [23]. The review also highlights specific barriers that need to be addressed, including failure of health workers to educate and invite TB patients to bring their close contacts to be screened, suboptimal processes and patient flows, TB-related stigma and knowledge gaps among HCWs, discrimination in contact tracing in the Budget, insufficient resources and limited access to diagnostic services, and communication barriers [26].

In order to surmount the barriers impeding TB-ACF and to amplify its facilitators, the present study suggests a range of measures. Firstly, enhancing community access to health facilities with TB diagnostic services is recommended. Secondly, there is a need to scale up the number of health extension workers in rural and remote areas. Furthermore, active case finding through community outreach and door-to-door visitation should be implemented. Lastly, addressing stigma through health advocacy and awareness programs is crucial. Thirdly, it is imperative to address these challenges by means of providing adequate training to healthcare workers [61].

Additionally, this review stresses the significance of improving access to care, reducing costs associated with seeking care, strengthening primary health-care services, providing outreach services for vulnerable populations, and implementing social protection schemes where necessary. These findings are in line with other studies conducted in LMICs, which have also adopted multiple interventions and strategies to enhance TB-ACF. However, more comprehensive and coordinated efforts are required to address the identified barriers and utilize the facilitators of TB-ACF effectively.

### The strength and limitation of the study

Active case finding represents the most prominent approach in controlling and preventing tuberculosis. Given its significance, this finding offers a critical source of information in the global efforts to eradicate TB, as it constitutes the first summary of active case finding. However, it should be noted that these findings cannot be universally applied across all populations and diseases. The strength of this systematic review is rooted in the use of different guidance and qualitative check tools, such as PRISMA, CEQual, BJI, and ENTREQ, thus underscoring its status as an evidence-based study. One of the limitations of this review is its small sample size, which only includes 23 studies, thereby potentially reducing its generalizability. Another limitation pertains to the analysis methods employed for the qualitative data, as the review relied solely on narrative summaries presented in tables and diagrams. The other basic limitation of this manuscript is lack of PROSPERO Registration number.

## Conclusion

This qualitative systematic review examines that stigma is the top patient- and community-related barrier, followed by health-system-related barriers such as lack of resources, including lack of diagnostic equipment, reagents, and consumables for the detection of cases with active TB. In this review, supervision, health worker training, leadership, integration into health systems, and long-term funding are key to the sustainability of tuberculosis-ACF. Financial support for staff time and program costs associated with expanding patient enrollments were seen as important factors in maintaining public sector commitment. In addition, government leadership, the existence of specific policies, the knowledge, clinical skills, and capacity building of frontline health workers are also key enablers for ACF in LMICs. The risks of stigmatization should be carefully assessed prior to initiating screening of the patients.

In conclusion, this review provides valuable evidence to support the modification and development of policies related to TB-ACF in LMICs. By addressing the identified barriers, promoting community engagement, and implementing bidirectional screening, policymakers can enhance TB case detection, reduce delays in diagnosis, and improve overall outcomes of TB-ACF programs.

### Electronic supplementary material

Below is the link to the electronic supplementary material.

Supplementary Material 1

## Data Availability

All important data and results are included in this manuscript.

## Abbreviations

ACF: Active Case Finding
JBI: Joanna Briggs Institute’s
ENTREQ: Enhancing Transparency in Reporting the Synthesis of Qualitative Research
CERQual: Confidence of Evidence Review Quality
PROSPERO: International Prospective Register of Systematic Reviews
CRD: Central Registration Depository
PRISMA: Preferred Reporting Items for Systematic Reviews and Meta-analysis
LHW: Lay Health Worker
HW: Health Worker
ASHA: Accredited Social Health Activists
CBNAAT: Catridege Based Nucleic Acid Amplification Test
