# Supplementary material for: Facilitators and barriers to tuberculosis active case findings in low- and middle-income countries: a systematic review of qualitative research
Source: BMC Infect Dis. 2023 Aug 7;23:515. doi: 10.1186/s12879-023-08502-7 (PMC10405492; doi:10.1186/s12879-023-08502-7)
Supplement: Supplementary file 1 — Supplementary Material 1 [file 12879_2023_8502_MOESM1_ESM.docx]

Additional file 1: Joanna Briggs Institute’s (JBI).

Reviewer ______________________________________ Date_______________________________ Record Number_________

**Author**: Melkie Dagnaw Fenta: **Year**: October, 2022

|  | Yes | No | Unclear | Not applicable |
| --- | --- | --- | --- | --- |
| 1. Is the review question clearly and explicitly stated? |  | □ | □ | □ |
| 1. Were the inclusion criteria appropriate for the review question? |  | □ | □ | □ |
| 1. Was the search strategy appropriate? |  | □ | □ | □ |
| 1. Were the sources and resources used to search for studies adequate? |  | □ | □ | □ |
| 1. Were the criteria for appraising studies appropriate? |  | □ | □ | □ |
| 1. Was critical appraisal conducted by two or more reviewers independently? |  | □ | □ | □ |
| 1. Were there methods to minimize errors in data extraction? |  | □ | □ | □ |
| 1. Were the methods used to combine studies appropriate? |  | □ | □ | □ |
| 1. Was the likelihood of publication bias assessed? | □ | □ | □ |  |
| 1. Were recommendations for policy and/or practice supported by the reported data? |  | □ | □ | □ |
| 1. Were the specific directives for new research appropriate? | □ | □ | □ |  |

Overall appraisal: Include **□** Exclude □ Seek further info □

Note: The answers were ticked in the given alternatives

**Addittional file- 2:** Enhancing Transparency in Reporting the Synthesis of Qualitative Research (ENTREQ).

| **Item** | **Guide and description** | **Reported on** |
| --- | --- | --- |
|  |  | **page #** |
|  |  |  |
| Aim | State the research question the synthesis addresses | 3 |
|  |  |  |
| Synthesis | Identify the synthesis methodology or theoretical | 4 |
| methodology | framework which underpins the synthesis, and describe |  |
|  | the rationale for choice of methodology (e.g. meta- |  |
|  | ethnography, thematic synthesis, critical interpretive |  |
|  | synthesis, grounded theory synthesis, realist synthesis, |  |
|  | Meta-aggregation, meta-study, framework synthesis). |  |
|  |  |  |
| Approach to | Indicate whether the search was pre-planned |  |
| searching | (comprehensive search strategies to seek all available |  |
|  | studies) or iterative (to seek all available concepts until | 4 |
|  | Theoretical saturation is achieved). |  |
|  |  |  |
| Inclusion | Specify the inclusion/exclusion criteria (e.g. in terms of | 5 |
| criteria | population, language, year limits, type of publication, |  |
|  | Study type). |  |
|  |  |  |
| Data sources | Describe the information sources used (e.g. electronic | 4 |
|  | databases (MEDLINE, EMBASE, CINAHL, psychINFO, |  |
|  | Econlit), grey literature databases (digital thesis, policy |  |
|  | reports), relevant organisational websites, experts, |  |
|  | information specialists, generic web searches (Google |  |
|  | Scholar), hand searching, reference lists) and when the |  |
|  | searches were conducted; provide the rationale for |  |
|  | using the data sources. |  |
|  |  |  |
| Electronic | Describe the literature search (e.g. provide electronic | 5 |
| Search | search strategies with population terms, clinical or |  |
| strategy | health topic terms, experiential or social phenomena |  |
|  | related terms, filters for qualitative research and search |  |
|  | Limits). |  |
|  |  |  |
| Study | Describe the process of study screening and sifting | 6 |
| screening | (e.g. title, abstract and full text review, number of |  |
| methods | independent reviewers who screened studies) |  |
|  |  |  |
| Study | Present the characteristics of the included studies (e.g. |  |
| characteristics | year of publication, country, population, number of | 7-8 |
|  | participants, data collection, methodology, analysis, |  |
|  | Research questions). |  |
|  |  |  |
| Study | Identify the number of studies screened and provide |  |
| selection | reasons for study exclusion (e.g. for comprehensive | 6 |
| results | searching, provide numbers of studies screened and |  |
|  | reasons for exclusion indicated in a figure/flowchart; for |  |
|  | iterative searching describe reasons for study exclusion |  |
|  | and inclusion based on modifications to the research |  |
|  | Question and/or contribution to theory development). |  |
|  |  |  |

**Additional file -3**: Medline search engine

1. ‘Tuberculosis/diagnosis’ OR ‘Tuberculosis/examination’ [Mesh]

2. ‘Tuberculosis OR TB OR Mycobacterium [Mesh]

3. 1or 2

4. ‘Active casefinding’ or ACF [Title/Abstract]

5. 3 and 4

6. ‘Qualitative Research’ [MeSH] OR ‘Interviews as Topic’ [MeSH]

7. ‘Qualitative or survey or ‘focus group*’ or interview* or questionnaire or experience* or ‘mixed method*” [Title/Abstract]

8. 6 or 7

9. 5 and 8

10. Barrier* or delay* or limit* or drawback*or challeng* or failure* or constrain* or [Title/Abstract]

11. 9 and 10

12. Facilitate* or enable*[Title/Abstract]

13. 11 and 12

14. Low income countries* or poor*[Title/Abstract]

15. 13 and 14

**Additional file -**4: PubMed search engine

((((((((("Facilitator**s**"[Mesh]) OR"enablers"[Mesh]) AND"barrier"[Mesh]) OR "delay"[Mesh])) OR “limit"[Mesh]) AND "Active casefinding" [Mesh]) OR "ACF "[Mesh]) AND"Tuberculosis"[Mesh]) OR "TB"[Mesh]) OR "Mycobacterium"[Mesh] AND "Human"[Mesh]) OR "People"[Mesh]) AND “Low income"[Mesh] AND"Middle income"[Mesh] AND"Countries"[Mesh])
